# Quest for cardiovascular interventions: precise modeling and 3D printing of heart valves

**DOI:** 10.1186/s13036-018-0132-5

**Published:** 2019-02-06

**Authors:** Rajat Vashistha, Prasoon Kumar, Arun Kumar Dangi, Naveen Sharma, Deepak Chhabra, Pratyoosh Shukla

**Affiliations:** 1 Optimization and Mechatronics Laboratory, Department of Mechanical Engineering, University Institute of Engineering and Technology, Maharshi Dayanand University, Rohtak, Haryana India; 2 Department of Medical Devices, National Institute of Pharmaceutical Education and Research Ahmadabad, Gandhinagar, Gujarat 382355 India; 3 Independent Researcher, Rohtak, 124001 India; 4 Department of Cardiology, Shalby Hospitals, Jabalpur, India; 5 Enzyme Technology and Protein Bioinformatics Laboratory, Department of Microbiology, Maharshi Dayanand University, Rohtak, Haryana 124001 India

**Keywords:** Cardiovascular fluid mechanics, Image processing, Biomaterials, 3D bioprinting, Mechanobiology

## Abstract

Digitalization of health care practices is substantially manifesting itself as an effective tool to diagnose and rectify complex cardiovascular abnormalities. For cardiovascular abnormalities, precise non-invasive imaging interventions are being used to develop patient specific diagnosis and surgical planning. Concurrently, pre surgical 3D simulation and computational modeling are aiding in the effective surgery and understanding of valve biomechanics, respectively. Consequently, 3D printing of patient specific valves that can mimic the original one will become an effective outbreak for valvular problems. Printing of these patient-specific tissues or organ components is becoming a viable option owing to the advances in biomaterials and additive manufacturing techniques. These additive manufacturing techniques are receiving a full-fledged support from burgeoning field of computational fluid dynamics, digital image processing, artificial intelligence, and continuum mechanics during their optimization and implementation. Further, studies at cellular and molecular biomechanics have enriched our understanding of biomechanical factors resulting in valvular heart diseases. Hence, the knowledge generated can guide us during the design and synthesis of biomaterials to develop superior extra cellular matrix, mimicking materials that can be used as a bioink for 3D printing of organs and tissues. With this notion, we have reviewed current opportunities and challenges in the diagnosis and treatment of heart valve abnormalities through patient-specific valve design via tissue engineering and 3D bioprinting. These valves can replace diseased valves by preserving homogeneity and individuality of the patients.

## Background

Aortic regurgitation, aortic stenosis, primary mitral regurgitation, secondary mitral regurgitation, mitral stenosis, tricuspid regurgitation, tricuspid stenosis along with coronary artery disease, rheumatic fever and bacterial endocarditis are among the most dampen factors, which leads to the valvular heart diseases (VHDs) and valve abnormalities [1, 2]. The penultimate prophylaxis for VHDs is by identification of disorders at an early stage. It enables prevention of usage of available end stage diagnosis/treatment procedures, i.e. prosthetic valve replacement via transcatheterization or surgery [3]. Besides, there also exist exclusive clinical guidelines issued by the eminent cardiologists concerning proactive steps for diagnosis and post/preoperative conditions at different stages along with the precise causes of these VHDs [4, 5].

These VHDs are associated with significant morbidity and mortality in an aged population, as they are correlated with vascular disorders. Considering the reasonable percentage of aged population in Europe, North America, Japan and other countries, VHDs are one of the prominent causes of death in these regions and need immediate attention [6]. In VHDs, the valves become either too contracted to open-up entirely or incapable to close effectively. In such cases, the diseased valves drive the blood in a reverse direction (i.e. adjacent heart chamber) while an incompetent valve results in blood leakage into the chamber into which it previously exited [7]. As a compensation for this inefficient pumping, the heart muscle enlarges and thickens, thereby losing its elasticity and morphology. These changes may result in hypoxic conditions leading to myocardial infarction, another fatal medical condition. Therefore, prosthetic valve replacement (via mechanical or biological valve) is the only exclusive solution available to compensate for the original valve under these rigorous circumstances [8]. Nevertheless, this treatment entails some malfunctioning such as leaking, excessive care, medication and frequent clinical follow-up through imaging [9]. Thus, it necessitates the search for an effective alternative such as patient-specific 3D printed, tissue-engineered valves using scaffolds and materials, which mimic the original valves [10, 11]. However, due to the limitations in scientific literature concerning the epidemiology, pathophysiology, mechanisms associated with regurgitation, stenosis and clinical management of VHDs, the development of alternative treatments for VHDs is quite challenging [12].

Therefore, the quest for contemporary interventions of VHDs requires a multidisciplinary, holistic approach where the amalgamation of inputs from engineering, medicine and basic sciences can generate a better understanding of VHDs to develop improved, patient-complaint pre/postoperative treatment and prevention methodologies. Hence, this review provides various contemporary opportunities along with the associated challenges for cardiac interventions to combat against such medical conditions. It also discusses the advances in biomaterials with suitable fabrication techniques which hold a promise to present recent advances in vascularized constructs, myocardium and heart valve conduits.

### Imaging interventions

The evaluation of cardiac condition is a pre-requisite for elucidation of the extent of severity and the level of medical emergency. In case of VHD and related disorders, it can be diagnosed through any of two methods: first line of investigation includes non-invasive imaging modalities such as echocardiography, stress testing, cardiac magnetic resonance, computed tomography and cinefluoroscopy, while the latter is conducted through invasive imaging techniques comprising of cardiac catheterization (IOCT, IVUS) and coronary angiography [13–15]. These invasive techniques are more prone to initiate medical complications such as ventricular septal defect, coarctation of aorta, and many others. Therefore, it requires a higher expertise and skillful hands to perform invasive imaging. Moreover, there also lies the associated risks such bleeding, infection, clotting with an invasive modality due to the contact of the vascular layer with a foreign material and medications that might restricts the use of invasive techniques within their own sense of operations. Therefore, significant decision making parameters confirming the existence of VHD through non-invasive investigations is essential to study that are depicted in the Table 1.

**Table 1:** Decision making parameters confirming the existence of VHD for most effective non-invasive investigations. ST = stress testing, CT = computer tomography, ECHO = Echocardiography, EROR = Effective Regurgitant Orifice Area (in mm^2^); RV = Regurgitant Volume (in ml/beat); MVPG = Mean Valve Pressure Gradient (in mm of Hg)

Intervention	AR	AS	PMR	SMR	MS	TR	TS	References
ST	Change in ejection fraction and stroke volume	MVPG > = 18	Quantitative methods for LV dysfunction	Quantitative methods for LV dysfunction	MVPG > = 10	–	MVPG > = 5	[60]
CT	Valve cusp characteristic	Degree of valve calcification and diminished aortic valve area	Thickened leaflet > 5 mm	–	Degree of valve calcification	–	–	[61]
ECHO	EROR > = 30; RV > =60	Degree of valve calcification	EROR > = 40; RV > =60	EROR > = 20; RV > =30	Valve area using planimetry	EROR > = 40; RV > =45	MVPG > = 5	[62, 63]

**Fig. 1:**
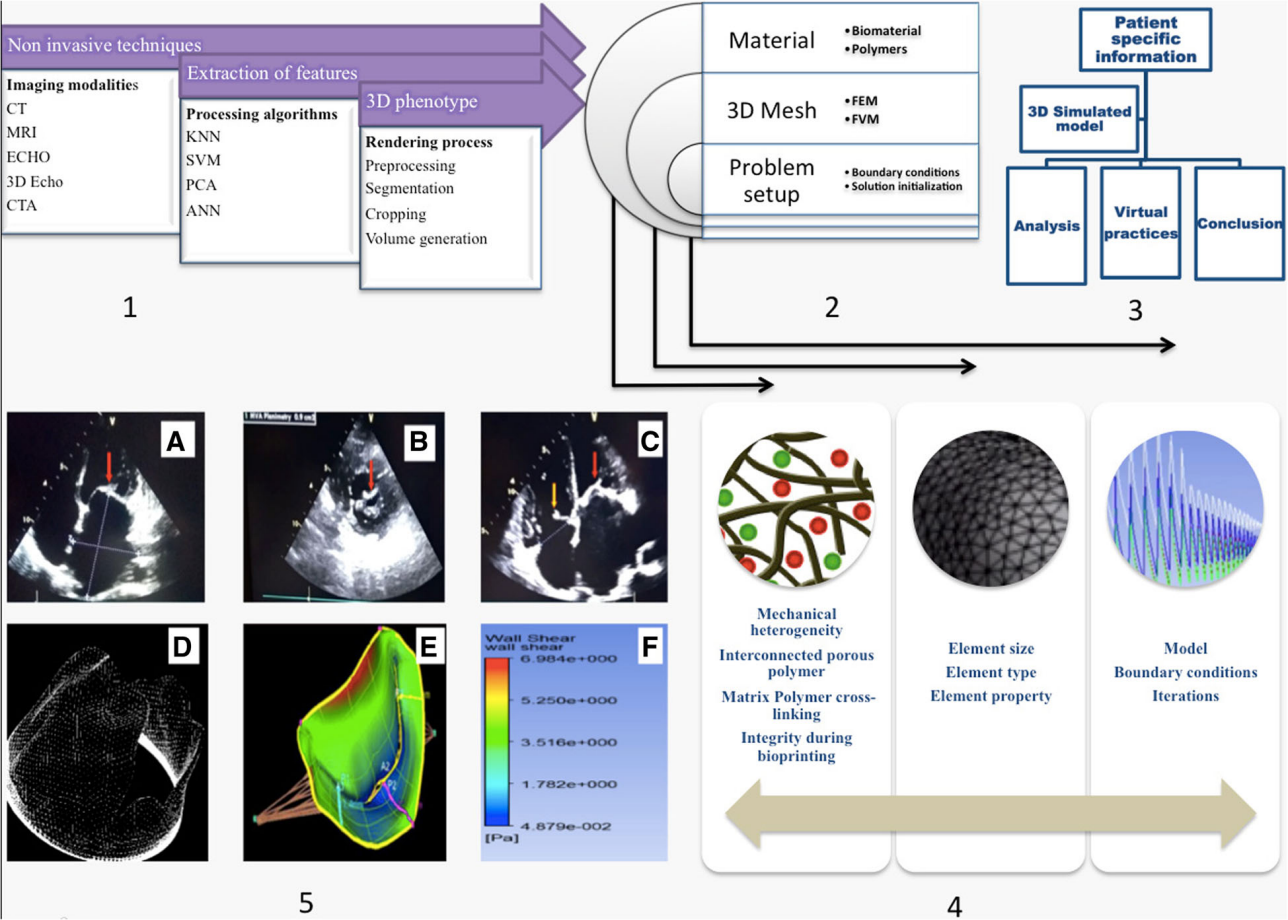
Schematic representation of the cardiovascular modeling process for patient specific diseases diagnostics. Processes 1, 2 and 3 show the sequential steps whereas step 4 and 5 shows conditions for real time processing. **a**. Thick and calcific Mitral valve with decreased opening in case of Chronic Rheumatic Heart Disease, (**b**). Parasternal Short Axis view of Mitral valve showing thickened anterior and posterior leaflets with reduced valve area, (**c**). Four Chamber view showing thickened Tricuspid Valve (yellow arrow) suggestive of organic Tricuspid valve disease and thick and calcific Mitral valve (red arrow) in case of Rheumatic Heart Disease, (**d**). 3D mesh for the volume generated geometry. **e**. Numerical setup for the problem in CFD software, F. Result post processing.)

However, there exists certain clinical cases that deviates from the standard categorization, hence requires operator preparedness to modify the imaging prognosis to effectively define the lesion in a heart valve. Precautious modeling, 3D volume rendering (VR) and curved multiplanar reconstruction (CMPR) are among these that are practiced during the modified diagnosis strategy. VR and CMPR enable higher vessel visualization and helps in preparing for patient specific surgical planning and intervention. There also exits digital imaging modalities using flat panel detectors on a rotating arm around the patient to generate volume data set that can be used on the parallel workstation to cast real time 3D angiography or CT imaging [16]. Integrated multidisciplinary approach for real-time 3D monitoring is shown in Fig. 1.

Another intervention that provides near field and far-field imaging with minimal interference and devoid of anesthetic procedure is minimally invasive intracardiac echocardiography (ICE). The ICE utilizes the intracardiac ultrasound imaging catheter to perform its function. Depending on the specific clinical case requirements, the physician can easily manipulate the ICE setup to intervene and employ it for an effective diagnosis purpose [17]. This radiation-free diagnosis can be a potential alternative to fluoroscopy imaging that exposes patients to the radiation. Provided the functionalities of ICE, Kenny et al., has discussed the commercially available ICE imaging techniques with their advantages and disadvantages [18]. The data obtained from above imaging techniques is used to develop preoperative simulated model for the physician. These phantom models are used for training surgical procedure before actual operative practice. A case history of a 69 year old patient, having a compromised mitral valve-in-ring, suggested successful implementation of diagnostic imaging techniques to acquire data, develop a simulated model for visualization and a preoperative patient-specific 3D-printed phantom model to predict adequate clearance via simulation. Further, fruitful transcatheter mitral valve-in-ring (TMV-in-R) was substituted after gaining input from preoperative procedures. This procedure produced excellent clinical (proper functioning of mechanical valve) and hemodynamic results [19]. Although it was clinical success, such interventions only provided a clinical tool to address the problem of stenosis or regurgitation. The underlying biomechanics of stenosis or regurgitation phenomena is least understood that might result in future complications like graft rejection, inflammation, delamination with nearby tissues and others. Therefore, mechanical interaction of blood and cardiac cells in such a mechanistic stressful environment of a valve need to be understood to ensure a successful integration of transplanted heart valve with a host tissue. Thus, these understandings are perquisite for the development of tissue-engineered valves and its effective remodeling and integration with native tissue without any physical abnormalities like changes in the mechanosensitive channels such as kinase due to hemodynamic pressure.

### Mechanobiology of stenosis

**Fig. 2:**
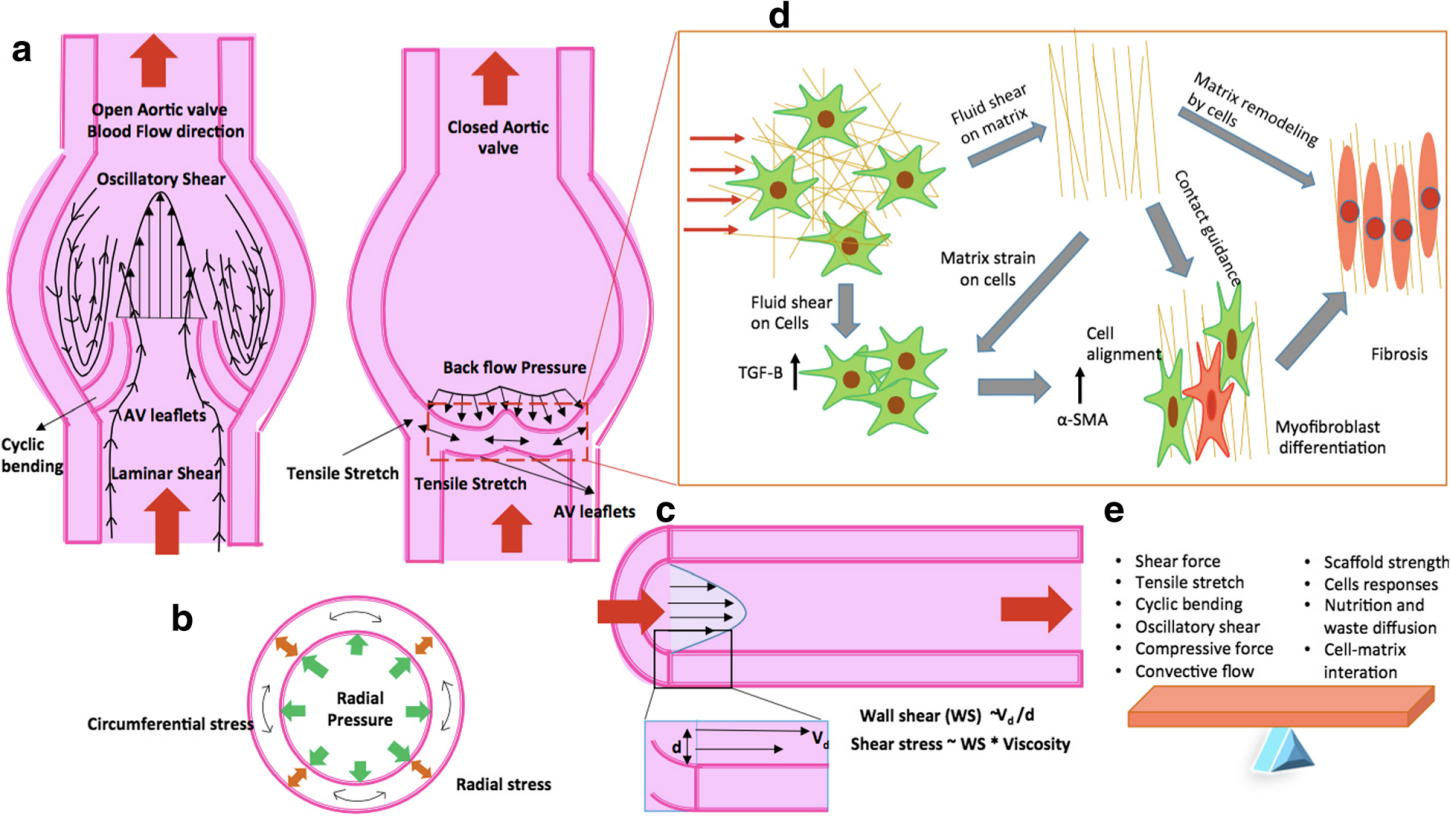
Schematic representation of the forces acting on the aortic valve during pulsatile blood flow and remodeling of fibrous matrix by cells of aortic valve under the influence of blood shear for open and closed state along with the factors responsible for the balanced state (**a**) representation of valve in frontal plane, (**b**) transverse cross-section view of blood vessel, (**c**) longitudinal cross-section view of the blood vessel, (**d**) fibrous matrix remodeling by cells and (**e**) balancing of factors while developing TEHVs

Studies demonstrated that mechanical forces play a significant role in VHD by the active control of the function of a valve at a cellular level. The schematic of aortic valve in a transverse and longitudinal cross-section illustrate the type of mechanical forces acting on the different regions of heart valve during its functioning (Fig. 2). The valvular endothelial cells (VECs) and interstitial cells (VICs) are sensitive to mechanical forces and interact with each other through shear forces (oscillatory shear and laminar shear) experienced during blood flow [20]. As the cyclic mechanical strain results in a tensile stretch in leaflets of heart valve during each stroke of pulsatile blood flow, this mechanical stimulus regulates the synthesis of glycosaminoglycans and proteoglycans by VECs and VICs. These biomolecules are instrumental extracellular matrix (ECM) remodeling in these tissues (Fig. 2a and d) [21]. Moreover, NOTCH signaling in VECs and paracrine signaling pathways in VICs being highly sensitive to shear stress, plays an important role in valve homeostasis or disease development. Further, VECs is also known to regulate the VICs phenotype and ECM synthesis [22]. It is likely that VECs experience differential shear stress at fibrosa-side of leaflets that increases the probability of rapid calcification for AVs. The changes in mechanical stresses result perturbation of the biosynthetic behavior of valve cells [23, 24]. The abnormal shear stress caused by ventricular infarctions in distinct regions, enhanced the collagen synthesis in the mitral valve leaflet that increases stiffness known as myxomatous remodeling [25]. On the other hand, abnormal hemodynamics flow and shear stress causes valve leaflets inflammation, including fibrous layer that leads to stenosis calcification or calcific aortic valve disease (CAVD) followed by valve failure [23]. Further, shear stresses up and down regulate the expression of proteins related to ECM, inflammation, osteogenesis and induced pluripotent stem cell (iPSC)-derived endothelial cells as described in Table 2.

**Table 2:** Mechanobiological effects on valve cells under various mechanical stresses

Mechanical stress	Markers	Mechanobiological effects	References
Shear stress	ECM proteins	↑ Collagen; ↑ MMP-2,9; ↑ TIMP-2; ↓ sGAG; ↓ cathepsin-L on ventricularis	[24, 64]
	Inflammation	↑ ICAM-1; VCAM-1 on fibrosa	
	Osteogenesis	↑ BMP-2,4; ↑ TGF-β on fibrosa; ↑ BAVs	
Pressure	ECM proteins	↑ Collagen; ↑ sGAG; ↑ β-catenin ↑ MMP-1,3; ↓ MMP-2,9; ↓ osteopontin	[24, 65]
	Inflammation	↑ VCAM-1; ↑ pentraxin-3; ↑ TNF-α; ↑ IL-6	
	Phenotype	↓ α-SMA	
Leaflet strain	ECM proteins	Elastin, ↑ MMP-1,2,9; ↑ collagen; ↑ cathepsin-S, K; ↓ TIMP-1; ↓ sGAG; ↓ cathepsin-L;	[24, 66]
	Inflammation	↑ ICAM-1; VCAM-1	
	Phenotype	↓ α-SMA	
	miRNA	↓ *miR-148a-3p*	

Furthermore, in the case of hypertension, valve cells experience high transvalvular pressures that lead to abnormal cyclic stretches, which increase the mechanical strains on the valve and accelerate the calcification [26]. As the calcification increases, the effective area around the valve reduced which further enhance the transvalvular pressure gradient and develop a positive feedback loop. Moreover, these abnormal cyclic stretches activate VICs to over express α-SMA is resulting more contractility and stiffness especially in CAVD [27]. Further, high pressure also up regulates and down regulates several cellular bio molecules that induces VECs alteration (Table 2), which ultimately remodel the ECM [24]. Along with shear stress and pressure, AV also undergoes anisotropic strain on their leaflet. The out-flow side; exert comprehensive stress while inflow-side experiences tensile stress (Fig. 2a, b and c). The leaflet strain also regulates the expression of markers involved in ECM inflammation, osteogenesis and phenotype (Table 2) through inducing VECs and VICs [24]. Moreover, recent studies also demonstrated the combined effect of different mechanical stresses in valvular pathophysiology. In this direction, Warnock and co-workers described the synergistic AV inflammatory response under combined cyclic strain and pressure than the individual response in the context of markers [28].

Thus, a complete understanding of mechanobiology of stenosis provides an insight into valve pathophysiology. However, these studies are limited due to the limitation on our probing capacity of a diseased valve at both cellular/molecular length scale and investigate their biomechanical manifestation on the disease and physiology of valves at macroscopic scale. Therefore, a diseased model of stenosis created using imaging and multi-scale computational modeling can provide an enriched insight of mechanobiology of stenosis.

### Mechanics for alternative therapeutics

Computational modeling and patient specific 3D simulations can be used to discover biological unfolds of VHD via assimilation of diverse data [29]. To optimize therapeutic parameters, analysis of flow and its effect on the four heart valves taking into account the patient anatomy, physiology and genetic information has been investigated using CFD [30]. However, the most significant parameters reviewed in this field are the relative high stresses with reference to geometric, microstructures, nonlinear and anisotropic constitutive behavior, loading conditions, along with kinematic constraint functions for the intervention of aortic and mitral valve abnormalities. Moreover, biophysical parameters with prevalent myocardial stiffness and contractility can be estimated by optimal matching of the behavior of these models to the data obtained from medical imaging. Thus, this integration provides new information on mechanisms of compensated and decompensated adaptation using less-invasive techniques [31]. Human heart simulator is formulated for the spatiotemporal evolution of electrical potentials and mechanical deformation across the heart using two-field finite element approach and Windkessel model [32]. It is used to probe landscapes of clinical parameters and guide device design for treatment planning in VHD. Based on this human heart simulator, an annuloplasty ring is designed that included a sub-valvular element to correct the valve dysfunction. This designing enables simulations of normal cardiac function as well as pathologic function in the setting of the posterior left ventricular papillary muscle infarction [33]. In another procedure, in vitro left heart simulator is used on which mitral valves were mounted and tested under pulsatile blood flow. This procedure quantifies anterior leaflet strain, leaflet coaptation length, depth, tenting area and regurgitation volume in the radial and circumferential directions at an increased level of geometric distortion [34]. The data generated from such studies provide a platform for the development of future surgical planning via computational modeling.

Replacing heart valves made-up from biologically derived materials is a holy grail of tissue engineering and these valves are referred to as bioprosthetic heart valves (BHVs). Although these valves have material and blood flow characteristics similar to the native valves, their failure continues to result from leaflet structural deterioration, mediated by fatigue or tissue mineralization. Therefore, to predict and optimize the distribution of stresses, tools from engineering are quite useful. The computational modeling through multi-scale mechanobiology under dynamic conditions was explored by Emmert et al., 2018 to determine the factors affecting the tissue remodeling by the seeded cells. Thus, knowledge gained was utilized to optimize the design of the tissue engineered heart valves (TEHVs). They demonstrated that computationally inspired design of TEHVs was better in tissue remodeling in sheep model [35]. Thus, these tools including quasi-static mechanics, dynamic structural mechanics, and more recently, fluid–structure interaction (FSI) can potentially provide better understanding of biomechanics of heart valve [36]. Immersogeometric FSI analysis is performed to parameterize the leaflet geometry using key design parameters (such as effective orifice area and the co-adaptation area) that is compared with patient-specific MRI data to demonstrate the qualitative similarity of the flow patterns in the ascending aorta. The imaging techniques combined with computational mechanics modeling have the potential to accelerate the design and development of bioprosthetic heart valves (BHVs). However, currently, clinicians opt for commercially available heart valves for their patients as TEHVs are currently in R&D stage. It is necessary to select an artificial heart valve from the pool of commercially available heart valves that demonstrates high matching scores when compared with the models generated from patient data.

In spite of such meticulous selection of heart valves, they suffer from regurgitation or stenosis, poor integration with host tissue, sometime immunological reactions and others. Moreover, there are hardly any alternative treatment modalities for fixing the partial defects or functional abnormalities in the native valves except the whole heart valve replacement. This unnecessarily increases the mortality and morbidity for patients. Henceforth advancements in imaging, computational modeling and designing tools need to be integrated with emerging areas of tissue engineering in order to develop human prosthesis similar to native tissues. Tissue engineering holds the potential to reduce patient–prosthesis mismatch in the direction of personalized medicine and accelerate the design and developmental time of prosthetic devices [37].

### Personalized 3D-printed cardiovascular prosthesis

Creating a construct of heart valve through tissue engineering principles can solve two purposes; first, the design and development of a bioartifical heart valve mimicking the structural and the functional aspect of a native valve that can be used as an implantable device and second, the generation of a disease model of a heart valve that can be used to generate understanding about the mechanobiology of the tissue and hence, assist in understanding a disease progressing, developing effecting therapeutic interventions. Before tissue engineering a heart valve, it is imperative to understand the multi-scale architecture, geometry and biomechanics of a heart valve’s parts that play a significant role in remodeling of a neo tissue matrix in the dynamic mechanical environment of a functional heart valve. These understandings will enable proper selection of biomaterials, fabrication methodologies, characterization tools and developmental environments for generation of tissue engineered heart valves (TEHVs).

The anatomy of the valves reveals that they are 3D structures experiencing a periodic physiological pressure of blood while entry and exit from a heart. The two major components of a heart valves are leaflets and root [38]. The leaflets are trilayer structures (fibrosa, spongiosa, and ventricularis) that continuously flaps, resulting in a closure and opening of a valve, experiences shear stress and periodic loading and unloading while the roots are annular structure whose one end provides support to the leaflet and its other end serves as a base to be integrated with the major blood vessels of a heart. Further, endothelial, smooth muscle, fibroblast and interstitial cells populate these valves in a defined spatial location having different flexure strength and stiffness. Provided the anisotropy in the mechanical and structural requirement and heterogeneity of cell types distribution, different biomaterials are needed to be used for the generation of different structures of a 3D heart valve having compatible biomechanical properties [39]. Further, the interface has to be a strong enough to prevent a damage at the inter junction of a leaf and a root.

Tissue engineering of heart valve through decellularised heart valves obtained from xenogenic or allogenic sources has been one of the best approaches [40]. The scaffolding results in an optimal micro environment for cells. However, this decellularization is achieved through several chemical and enzymatic treatments that weaken the scaffold, making it hostile to be further developed in a bioreactor [41]. Further, it also poses antigenic immunological reaction at the site of graft in patients. Therefore, usage of synthetic biomaterial for scaffold development has gained importance in recent years. The use of materials like polyhydroxyalkanoates, polyhydroxyoctanoates, polyglycolic acid, polylactic acid, chitosan, collagen, polyglycerolsebacatehas been used to create a porous scaffold mimicking the morphology and geometry of heart-valve through different microfabrication techniques like electrospinning, salt-leaching, hydrogels, cryogels and others [42–45]. These fibrous, porous scaffolds mimic the ECM, thereby provides adequate mechanical and topological cues for adhesion, migration, growth, differentiation and proliferation of cells. In addition, the porous structure also enabled easy exchange of nutrients and oxygen to the seeded cells and removal of metabolic waste from them [46]. In spite of such advantages, it has been observed that precursor cells from heart or blood enter in the porous scaffold during an implant and exhibits traits of thrombosis, contraction, osteogenic phenotype, mineralization, a hallmark of non-conducive micro environment for cells [47]. The design of these scaffolds severely limits the neo tissue remodeling by the seeded cells. The seeded valve cells either result in a thickened stenotic valve or hyper contracted valve that fails to close properly. This might be due to an inherent limitation of above methods to fabricate scaffolds with mechanical heterogeneity and asymmetry and provide mechanical cues with spatial variation. Further, it is challenging to create a monolithic, complete, complex geometry of heart-valve through the above methods, except its individual parts i.e. leaf, roots and others. In addition, achieving mechanical properties commensurate with spatial location is still a bottleneck with above fabrication methods. Therefore 3D printing/bioprinting has been explored for the heart-valve generation.

There are several 3D printing technologies that have a potential for create complex heart valve structures. The extrusion based 3D printing technologies uses either organic solvent or heat to create polymer solution or melt for generating 3D structures. Hence, they are hardly suitable for any live cell incorporation during printing except creating a 3D solid scaffold for seeding different cell types of a heart valve. The particle fusion based 3D printing is practiced either by bonding ceramic microparticles with any bioglue or by sintering metallic particles with high energy laser beam [48]. The ceramic based 3D printing may support the cells incorporation but its mechanical properties may not be in congruence with a natural heart valve. Further, the degradation product of ceramic creates acidic environment that is hostile for heart valve cells in a long run [48, 49]. The 3D printing based on metallic sintering is not suitable for heart value due incompatible mechanical properties and thermal pre and post processing. Similarly, SLA based 3D printing is limited due harsh nature of UV cross-linking, extensive post-processing and lack of biocompatible and biodegradable materials acceptable in SLA. Therefore, inkjet printing and bioprinting are the 3D printing technologies that utilizes cell laden droplet bioinks or hydrogels for 3D printing of tissues and cells [50–52].

**Fig. 3:**
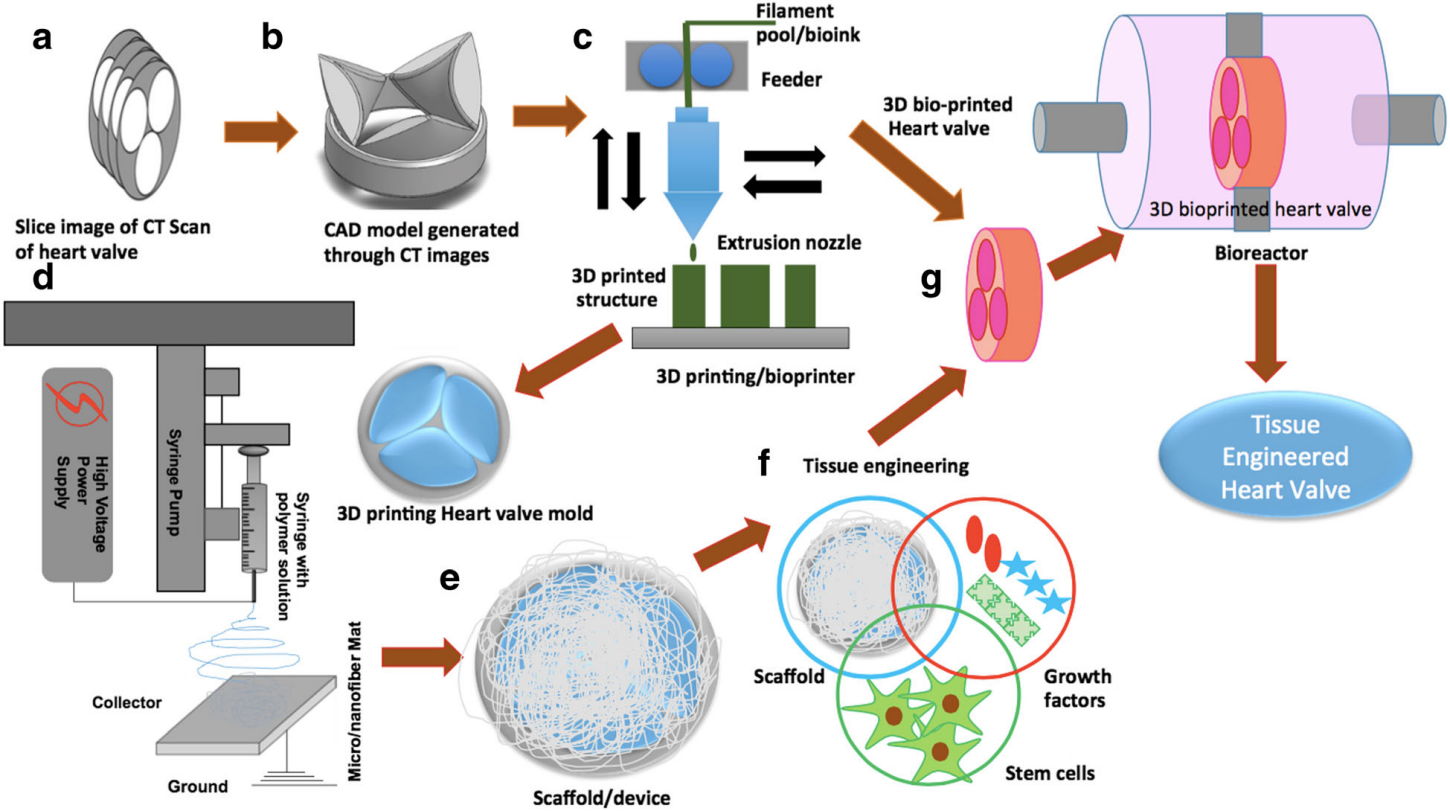
Schematic representation of the proposed process for the generation of 3D heart valves through combining either bioprinting or combination of 3D printing and electrospinning with bioreactor to arrive at functional tissue engineered heart valves (**a**) slice of CT images, (**b**)3D CAD model generation, (**c**) 3D bioprinting through bioink/ 3D printing through PLA, PCL materials, (**d**) combining PCL-Gel electrospun nanofibrous with 3D printed scaffold, (**e**) scaffold ready for conventional tissue engineering (**f**) Development of tissue through combining stem cells, growth factors and developed scaffold, (**g**) Development and initial tissue remodeling in perfusion bioreactor under dynamic pulsatile flow conditions

The heart of any bioprinting technique is a bioink, a gel material capable of being printed in a 3D architecture defined by a CAD model [53]. These CAD are either modeled through processing of CT, MRI images by volume rendering and modeling techniques (Fig. 3) or through computationally inspired designed as described by Emmert et al., 2018 [35]. The several hydrogels have gained popularity as a bio-ink, listed in the Table 3 [54–56]. The biomaterials like synthetic polymers (e.g., PEG, PEGDA), natural polymers (e.g.hyaluronic acid) and protein materials (e.g., fibrin, collagen, and glycosaminoglycans) are the best sources to produce hydrogels [54]. Further, the strength of the hydrogels can be tailored by variation in the cross-linking among the polymer chains. This can be achieved well by changes in the UV exposure time, cross linker density, temperature and others during the fabrication process. Moreover, 3D bioprinting also facilitates seeding different cell types at different spatial locations mimicking heart valve tissue during 3D bioprinting [56]. However, considering the regional and spatial mechanical heterogeneity of a heart valve, it is still a challenge to achieve completely controlled regional and spatial cross-linking in 3D printed hydrogels. The changes in the mechanical properties of gel must be carried out without affecting the physiological behavior of the different types of seeded cells (Table 3) in the spatially different regions of the heart valve. For example, excessive UV exposure or cross linker may be toxic to cells, excessive mechanical strength in the gel may lead to different phenotypic expression by cells and lower strength will make the device hostile to be further used in bioreactors [55, 57]. The increase in the thickness of the 3D bioprinted tissue poses a serious limitation on the transport of gases, nutrients and waste to and from the cells residing in the core of the 3D printed tissue. This results in a hypoxic and nutrient deficient center. Thus, there is need for the development of vascularization during the fabrication process. Moreover, bioprinting of composites of electrospun nanofibers and hydrogels can be a used for localized enhancement of mechanical strength. This will not only maintain the mechanical integrity of the scaffold but also provide mechanical cues necessary for precursor cells to differentiate into VICs, VECs and other cell types during static/dynamic cell culture. However, there has to be a balance between the amount of polymers and cells, such that polymers can provide enough mechanical strength to the scaffold while cell grows happily during tissue development and remodeling. We propose a combination of different fabrication technologies discussed above to arrive at scaffolds having spatial mechanical heterogeneity, 3D multiscale architecture and physiochemical characteristics that can facilitate appropriate growth, development and remodeling attributes of resident valve cells. These developed scaffolds can be further developed by providing biomechanical stimulus in perfusion bioreactors (Fig. 3).

**Table 3:** Bio-ink and cell types to be used in 3D bioprinting of heart valve

Bio-ink for 3D bioprinting	Hydrogel	Natural polymers like, agar, gelatin, collagen, cellulose, fibrinogen, hyaluronic acid, or from synthetic polymers such as polyacrylamide, alginate,polyurethane, poly-ethylene-glycol or synthetic-natural mixtures like gelatin methacrylamide (GelMa), Matrigel and mixed Pluronic and calcium phosphate cell-laden hydrogels, two component DNA hydrogel ink system, poly(N-(2-hydroxypropyl) methacrylamide lactate) A-blocks partially functionalized with methacrylate groups, and poly (ethylene glycol) B-blocks, Methacrylated hyaluronic acid (MeHA) macromers were either modified with adamantine (Ad-MeHA) or β-cyclodextrin (CD-MeHA), nanocellulose-based bioink like Nano-fibrillated cellulose (NFC) + alginate,	
	Ceramic hydrogel composite	poly (vinyl alcohol) (PVA) and alginate combined with bioactive glass and dexamethasone, hyaluronic acid combined with UV-curable glycidyl methacrylate, metal powders mixed with polylactic-co-glycolic acid (PLGA) in DCM, PVA and phytagel (1:1), Nano-fibrillated cellulose (NFC) + Hydroxyapatite (HA)	
Cell used in heart valve tissue engineering	Animal source	Cells	Mesenchymal stem cell, Valvular interstitial cell, Valvular endothelial cell, Endothelial progenitor cell, Endothelial cell, Bone marrow progenitor cell, Autologous amniotic fluid cell, Smooth muscle cell, Myofibroblast
		Tissue/animal	Bone marrow/bovine, Aortic valve/porcine, Aortic valve/bovine, Peripheral blood/sheep, Carotid artery/lamb, Bone marrow/lamb, Amniotic fluid/sheep, Aortic root sinus/porcine, Aortic wall/porcine
	Human source	Cells	Mesenchymal stem cell, Endothelial progenitor cell, Valvular interstitial cell, Induced pluripotent stem cell
		Tissue	Bone marrow, Adipose tissue, Umbilical cord matrix, Umbilical cord blood, Amniotic fluid, Chorionic villi, Amniotic fluid, Peripheral blood, Umbilical cord blood, Aortic valve, Skin fibroblasts

Achieving regional and spatial heterogeneity in the mechanical properties in complex 3D architecture might be a daunting task; however, it is relatively feasible to achieve regional heterogeneity in mechanical properties in a planner device made up of biocompatible hydrogels. 4D printing has allowed the researchers to codify different mechanical stiffness at different locations in planner object made up of stimuli responsive materials [58]. The heterogeneity in the Young’s modulus of hydrogels can be achieved through the introduction of fibrils during printing in a planar device. Due to the variation in stiffness of the materials and its swelling behavior, these modified hydrogels get folded in 3D architecture on exposure to biofluids [59]. Thus stimuli responsive biomaterials can be printed and folded in 3D complex geometries upon stimulation with the appropriate environment. Thus, there exist possibilities to develop heart-valve through origami inspired folding of planner biomaterial into complex 3D heart valve geometry having appropriate spatial mechanical properties. Although, none of the tissue engineering approaches till now have been successful in generating fully functional heart valve, they have their own advantages and disadvantages in heart valve regeneration as listed in the Table 4. We believe that novel biomaterials supporting spatio-temporal control over the mechanical properties during 3D bioprinting can lead to a development of patient specific bio artificial heart valves.

**Table 4:** Table for comparison between advanced materials and traditional materials

Scaffolding Processes	Materials	Advantages	Disadvantages	References
Decellularization of Allogenic /Xenogenic tissues	Heart valve obtained from Allogenic /Xenogenic sources	Easy to develop, resembles geometry of the native heart valve, biocompatible	Loss of mechanical anisotropy due to erosion, antigenic reactions during transplant, lacks strength to be developed in bioreactors	[41]
Electrospinning, salt leaching	polyhydroxyalkanoates, polyhydroxyoctanoates, polyglycolic acid, polylactic acid, chitosan, collagen, polyglycerol sebacate, polycaprolactone, Chitosan, HAP, fibronectin, HA, PEG, PNIAAm, PAA, PMMA, PAam, and PDMAEM	Fibrous, porous scaffold mimicking ECM, ability to form simple 3D structures, cells gets adequate bio-mechanical cues for growth and development, nutrients and waste exchange is better	Lacks elastomeric property as a native valve, inability to tailor spatial heterogenity in mechanical properties of scaffolds, inability to form 3D complex geometry of valves, sometime leads to thrombogenecity, non-conducive environment for cells	[42–45]
Bioprinting	Self-assembling elastomeric peptide materials, alginate-gelatin hydrogels, fibroblast-laden fibrin gel, Protein-based hydrogels, methacrylated hyaluronic acid, methacrylated gelatin, combination of 700 and 8000 MW poly (ethylene glycol) diacrylate (PEGDA), collagen, hyaluronic acid	Easy to fabricate 3D complex geometries of heart valve, ability to tailor the stiffness of materials during bioprinting, cells experiences microenvironment suitable for growth and development	Difficulty in printing a large structure, Structurally weak materials after printing, challenges in further developing the tissue through 3D printed structure in bioreactor	[54, 55, 57]
4D printing	biopolymers (alginate and hyaluronic acid), thermo responsive polymers,	Control over the spatial material stiffness, ability to obtain 3D geometries on appropriate stimulation	A nascent technology with very few material compatibility, challenges in codifying different regional and spatial mechanical properties for folding in 3D shape upon stimulus	[58, 59]

## Conclusion

The underlying motivation to investigate patient-specific valve printing via tissue engineering and 3D bioprinting is to find significant causes and conclude measures to eradicate potential defects in prosthetic heart valves. In this review, we attempted to describe the possible engineering tools and techniques for better diagnosis of stenosis, understand the underlying biomechanics at a different length scale, proper treatment planning and practices and finally the potential pitfalls of the current clinical interventions. We has also discussed the role of futuristic technologies like bioprinting and 4D printing along with the possible biomaterials to develop bioprosthetic valves --- a step closer to personalized medicine. Moreover, some recent research undertakings signifying the use of 3D printing and scaffolds towards VHDs engineering is shown in the Table 5. Although this state of art is currently limited in its performance, nevertheless we propose a formal set of notion to overcome it.

**Table 5:** Recent research undertakings signifying use of 3D printing and use of scaffold towards VHDs

Year	Title of the work	Practice followed	Foremost Inferences	Reference
2018	Engineering a 3D-Bioprinted Model of Human Heart Valve Disease Using Nano indentation-Based Biomechanics	3D-bioprinted CAVD model is engineered and layer-specific mechanical properties of the human AV was studied.	It potentiates the micro calcification by mimicking the native AV mechanical environment	[67]
2018	Comparison of the two biological aortic valve prostheses inside patient-specific aorta model by bi-directional fluid-structure interaction	Reverse engineering is used to create a 3D CAD model for biological aortic valves prostheses	Fluid solid interaction Stress analyses of the leaflets showed two stresses peak within the initial 0.3 s	[68]
2018	Modeling conduit choice for valve-sparing aortic root replacement on the biomechanics with a 3D-printed heart simulator	Valsalva grafts deform the radial position of the aortic valve. It results in an impaired leaflet motion, higher stresses, and potentially reduced valve performance compared to straight tubular grafts.	Valsalva conduits may have damaging consequences on the valve performance	[69]
2018	Toward predictive modeling of catheter-based pulmonary valve replacement into native right ventricular outflow tracts	RVOT models created from pre-implant and post harmony valve implant CT scans. Further using a software, virtual transcatheter pulmonary valves (TPVs) is placed in a RVOT model	Pre-implant modeling that assumes a rigid vessel quite accurately predicts the degree of distal RVOT expansion following an actual device replacement.	[70]
2017	Computationally designed 3D printed self-expandable polymer stents with biodegradation capacity for minimally invasive heart valve implantation: A proof-of-concept study	A commercially available 3D printing polymer was selected, and crush and crimping tests were conducted to validate the results predicted by the computational model	It demonstrates the design and manufacturing of a polymer stent with a mechanical performance comparable to that of conventional nitinol stents used for heart valve implantation in animal trials	[71]
2017	Utility and scope of rapid prototyping in patients with complex muscular ventricular septal defects or double-outlet right ventricle: Does it alter management decisions?	Various imaging modalities are used to develop patient-specific anatomic models via rapid prototyping	Intra-cardiac anatomy in CHD is accurately defined using patient-specific 3D heart models	[72]
2017	3D printing based on cardiac CT assists anatomic visualization prior to transcatheter aortic valve replacement	Pre-TAVR cardiac computed tomography is used to develop 3D printed models of the aortic root	The physical interplay of the aortic root and implanted valves are assessed efficiently using Pre-TAVR 3D-printing	[73]
2017	A low-cost bioprosthetic semilunar valve for research, disease modelling and surgical training applications	Computer-aided design files are provided for making the frame from wire or by metal 3D printing	It demonstrate that the valves can replicate the performance of clinical valves for research and training purpose	[74]
2014	Three-dimensional printing in cardiac surgery and interventional cardiology: a single-centre experience	It represents case study of 3D printed models using preoperative computed tomography or MRI in pediatric and adult cardiac surgery.	3D printing models is likely for perioperative planning and simulation in a diverse complex cases for pediatric and adult cardiac surgery, as well as for interventional cardiology	[75]
2014	Three-dimensional printed trileaflet valve conduits using biological hydrogels and human valve interstitial cells	Based on methacrylated hyaluronic acid (Me-HA) and methacrylated gelatin (Me-Gel), 3-D printable formulations of hybrid hydrogels are developed. It is used to bioprint heart valve conduits containing encapsulated human aortic valvular interstitial cells (HAVIC)	The first rational design of bioprinted trileaflet valve hydrogels that regulate encapsulated human VIC behavior	[76]

## Abbreviations

3DTEE: Three-dimensional transesophageal imaging
AI: Artificial Intelligence
AV: Aortic valves
BHVs: Bioprosthetic heart valves
CAVD: Cardiovascular diseases
CFD: Computational fluid dynamics
CHD: Congenital heart diseases
CMPR: Curved multiplanar reconstruction
DIP: Digital image processing
ECM: Extra cellular matrix
ICE: Intracardiac echocardiography
iPSC: Induced pluripotent stem cell
TMV-in-R: Transcatheter mitral valve-in-ring
VEC: Valvular endothelial cells
VHD: Valvular heart disease
VICs: Valvular interstitial cells
